# Neutrophils in Type 1 Diabetes: Untangling the Intricate Web of Pathways and Hypothesis

**DOI:** 10.3390/biom15040505

**Published:** 2025-03-31

**Authors:** Laura Nigi, Erika Pedace, Francesco Dotta, Guido Sebastiani

**Affiliations:** 1 Diabetes Unit, Department of Medicine, Surgery and Neurosciences, University of Siena, 53100 Siena, Italy; laura.nigi@unisi.it (L.N.); erika.pedace@student.unisi.it (E.P.); guido.sebastiani@unisi.it (G.S.); 2 Fondazione Umberto Di Mario ONLUS, Toscana Life Sciences, 53100 Siena, Italy; 3 Tuscany Centre for Precision Medicine, 53100 Siena, Italy

**Keywords:** neutrophils, type 1 diabetes, innate immunity, autoimmune diseases

## Abstract

Neutrophils are increasingly recognized as key contributors to the pathogenesis of Type 1 Diabetes (T1D), yet their precise mechanistic role in disease onset and progression remains incompletely understood. While these innate immune cells reside in pancreatic tissue and support tissue homeostasis under physiological conditions, they can also drive tissue damage by triggering innate immune responses and modulating inflammation. Within the inflammatory milieu, neutrophils establish complex, bidirectional interactions with various immune cells, including macrophages, dendritic cells, natural killer cells, and lymphocytes. Once activated, they may enhance the innate immune response through direct or indirect crosstalk with immune cells, antigen presentation, and β-cell destruction or dysfunction. These mechanisms underscore the multifaceted and dynamic role of neutrophils in T1D, shaped by their intricate immunological interactions. Further research into the diverse functional capabilities of neutrophils is crucial for uncovering novel aspects of their involvement in T1D, potentially revealing new therapeutic targets to modulate disease progression.

## 1. Introduction: Neutrophils and Type 1 Diabetes

Neutrophils represent the most abundant circulating white blood cells in humans and are key components of the innate immune system as first line defenders against host-invading pathogens, including viruses, bacteria and fungi. They are known to not only circulate in the bloodstream but also to reside in various organs and tissues, including the pancreas, where they play a role in maintaining tissue homeostasis and contribute to essential physiological processes such as coagulation, angiogenesis, and tissue repair [1,2,3,4]. Additionally, resident neutrophils probably behave as sentinels, triggered by the local microenvironment to respond promptly to invading microorganisms [3,4].

Neutrophils are widely recognized for their ability to engage in complex communication with various cells, including both innate and adaptive immune cells, such as dendritic cells (DCs), monocytes, macrophages, T cells, B cells, and natural killer (NK) cells, as well as non-immune cells like platelets, epithelial cells, and endothelial cells. These interactions are pivotal in establishing the role of neutrophils in processes such as hemostasis, mucosal inflammation, and atherogenesis [5], while in influencing various aspects of the immune response and in both initiating and regulating inflammation [6,7,8].

In the pancreas, neutrophils appear to play a role in β-cells function and dysfunction. As a matter of fact, it is plausible that, through the secretion of cytokines, chemokines or other factors secretion, they may directly or indirectly influence β-cells function, thereby contributing to the islet’s response to metabolic demands [1].

On the other hand, the insulin receptor is constitutively expressed on the neutrophil plasma membrane [9,10], indicating that this hormone plays a role in regulating neutrophil metabolism. Additionally, insulin appears to regulate neutrophil protein production and contribute to the modulation of certain immune functions [11]. Under normal physiological glucose concentrations, it has been shown to stimulate neutrophil chemokinesis in healthy individuals and enhance their migration toward a positive gradient of chemotactic substances [12,13]. Moreover, insulin has been found to modulate the inflammatory response of neutrophils and play a role in regulating their phagocytic and bactericidal capacities [14].

Neutrophils can also contribute to tissue damage by priming innate or adaptive immune responses and modulating inflammation [15]. Supporting this, recent advancements in technology have shed light on the crucial role of neutrophils, as key players in the innate immune system, in initiating and sustaining autoimmune disorders, such as T1D. These findings suggest that these cells are far more versatile and heterogeneous than previously thought [3]. However, the mechanisms through which neutrophils interact with other immune cells in the context of autoimmune diseases remain poorly understood and warrant further investigation [16].

T1D is a chronic autoimmune condition characterized by the immune system’s targeted dysfunction and destruction of pancreatic β-cells, leading to insulin deficiency [17]. The primary agents responsible for β-cells destruction appear to be autoreactive CD8+ T lymphocytes; however the autoimmune response in T1D involves both the adaptive and the innate immune systems [18,19,20,21]. Within this context, the role of neutrophils in T1D pathogenesis has garnered a lot of attention as potential key players in the initial stages of the disease. In the landmark study by Diana et al. [22], it was observed that physiological β-cell death can trigger the recruitment and activation of B-1a cells, neutrophils, and plasmacytoid dendritic cells (pDCs) in the pancreas of young Non Obese Diabetic (NOD) mice. The presence of neutrophils and pDCs was transient, peaking at 3 and 4 weeks of age, respectively. The interaction between these cells was suggested to be essential for initiating autoimmune diabetes. Specifically, β-cell debris could form immune complexes with dsDNA-specific IgGs secreted by B-1a cells, while neutrophils might produce DNA-binding peptides that enhance these immune complexes and stimulate IFN-α secretion by pancreatic pDCs, via TLR9. In this process, neutrophils were identified as a source of CRAMP (the neutrophil granule protein cathelicidin) and appeared to release neutrophil extracellular traps (NETs), typically associated with CRAMP release and the activation of IFN-α–secreting pDCs. This highlights the role of CRAMP-secreting neutrophils in activating pancreatic pDCs and triggering the onset of T1D. Ultimately, this sequence of events creates an inflammatory environment conducive to an adaptive immune response driven by autoreactive T cells, culminating in the development of autoimmune diabetes. Still regarding NOD mice, the neutrophils recruitment inhibition, mediated by the CXCL8-CXCR1/2 pathway, is able to prevent and revert hyperglycemia and neutrophil neutralizing antibodies utilization in the preclinical stage of autoimmune diabetes could enhance disease progression [1,22,23]. In humans, pancreas-infiltrating neutrophils have been observed in T1D patients just before the clinical onset of the disease. These neutrophils were primarily located in very small blood vessels and in the exocrine pancreas, as revealed by electron microscopy and immunohistochemical analysis [1,24,25]. Moreover, in patients with recent-onset T1D, peripheral blood circulating neutrophils were observed to be reduced to the lower end of the normal range. Similarly, first-degree relatives of T1D patients with two or more autoantibodies against β-cell antigens exhibit reduced neutrophil counts, which correlate with the progression of β-cell dysfunction [24,26]. This neutrophil reduction does not appear to result from increased neutrophil death, impaired differentiation, or because of anti-neutrophil antibodies; instead, it suggests that before the clinical onset of T1D, neutrophils may move from bloodstream to the pancreas [24]. Neutrophils from both clinically manifest and pre-symptomatic T1D individuals exhibit an interferon (IFN)-driven pro-inflammatory signature [25], similar to patterns observed in other autoimmune diseases such as rheumatoid arthritis (RA) and systemic lupus erythematosus (SLE) [27,28,29]. Moreover, in T1D patients, neutrophils demonstrate altered functions, including reduced migration and chemotaxis and impaired phagocytic activity [26,30,31]. Levels of neutrophil elastase (NE) and proteinase 3 (PR3) serine proteases from neutrophil granules, involved in microbial clearance and immune regulation during inflammation, are elevated in T1D patients, as well as myeloperoxidase (MPO, an enzyme essential for microbial killing) [32] and correlate with titer and number of β-cell specific autoantibodies [33]. Enhanced NETs, web-like structures formed from cytosolic and granule proteins on decondensed chromatin and released by activated neutrophils to capture and eliminate pathogens [26,34], have been observed in the circulation of T1D patients [33]. These NETs display an altered composition compared to those of healthy individuals [26,35]. Particularly, peptidyl arginine deiminase-4 (PAD4), an enzyme required for histone citrullination and NET formation, is upregulated in T1D neutrophils, resulting in heightened NETosis upon stimulation [36]. Furthermore, pancreas-infiltrating neutrophils in T1D patients also release NETs, further implicating their role in the disease process, as in other autoimmune diseases like SLE and RA [6,26,29]. The precise mechanistic role of neutrophils in the onset and progression of T1D remains to be fully understood. However, it is crucial to note that during the development of pancreatic islet inflammation, there is a significant interaction between both adaptive and innate immune cells [37]. The presence of innate immune cells has been observed in both the exocrine and the endocrine pancreas during the early stages of insulitis [1,18]. In this innate inflammatory environment, neutrophils may interact intricately and bidirectionally with various immune cells, including macrophages, NK cells, DCs, and lymphocytes [1]. Once activated, neutrophils could enhance the innate immune response through different mechanisms such as degranulation, phagocytosis, reactive oxygen species (ROS) production, complement system activation, release of cytokines and NETs [1]. Recent findings suggest that neutrophils also have the capability to present antigens. Furthermore, different neutrophil subtypes have been identified, some with pro-inflammatory functions and others exhibiting immunosuppressive properties [3,7,8]. This suggests a potentially complex and diverse role for neutrophils in autoimmune diabetes. However, despite the renewed interest in this area, the data regarding the involvement of neutrophils in the pathogenesis of T1D, particularly in humans, remain limited. Several reviews in the literature specifically examine the role of neutrophils in the pathogenesis of T1D [6,26,38,39,40]. However, none have focused on comprehensively discussing the immunological interactions of neutrophils, which we believe are crucial for understanding their exact role in autoimmune diabetes. In this review, we will thoroughly examine the intricate interaction between neutrophils and other immune cells that could influence β-cell fate in T1D through the exploration of three main mechanistic hypotheses suggesting how neutrophils may play an active role in the disease’s pathogenesis: (i) direct or indirect interaction with other immune cells involved in T1D pathogenesis; (ii) islet-antigen presentation to the adaptive immune system; (iii) destruction or dysfunction of β-cells through the secretion of pro-inflammatory factors. Our final goal is to shed light onto novel and lesser-known aspects of these interactions, providing fresh insights that may encourage the scientific community to further investigate this critical area of research.

## 2. HYPOTHESIS-1: Neutrophils Engage Other Immune Cells Which in Turn Cause Dysfunction and Destruction of β-Cells

### 2.1. Direct Neutrophils-Immune Cells Interaction

**Figure 1:**
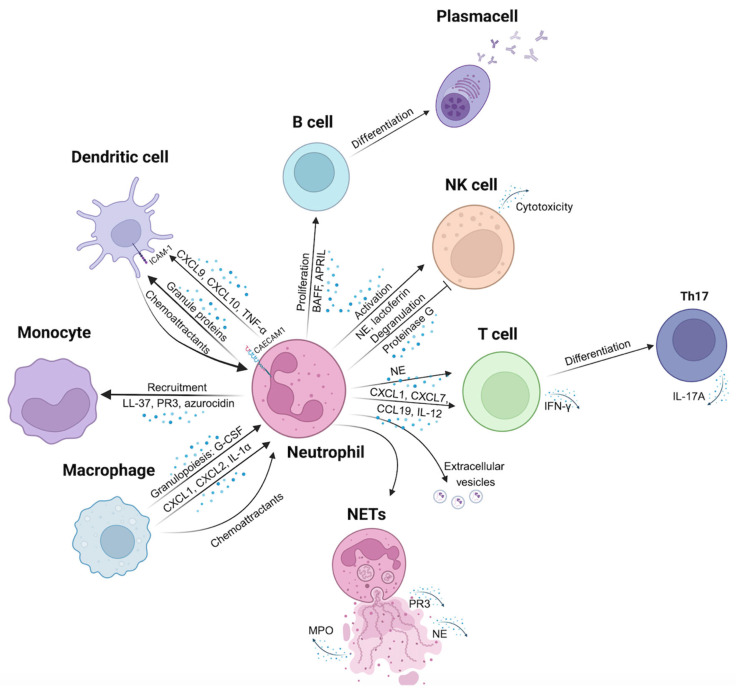
Hypothesis-1: Direct and remote communications among neutrophils and immune cells that lead to autoimmunity. Macrophages play a key role in regulating granulopoiesis by releasing G-CSF and secreting chemoattractant molecules like CXCL1, CXCL2, and IL-1α. Neutrophils recruit monocytes to inflamed tissue through the release of LL-37, PR3, and azurocidin. Communication with dendritic cells is mediated by interactions between CEACAM1 and ICAM-1, as well as the secretion of CXCL9, CXCL10, and TNF-α; furthermore, granule proteins secreted by neutrophils act as chemoattractants for dendritic cells. Neutrophils influence B cell activity by secreting BAFF and APRIL, that regulate B cell proliferation and differentiation. They activate NK cells through the secretion of NE and lactoferrin, while simultaneously inhibiting NK cell degranulation via proteinase G release. Neutrophils can promote T cell differentiation into Th17 cells through NE secretion; they also secrete CXCL1, CXCL7, CCL19, and IL-12, facilitating T cell recruitment. A key communication mechanism employed by neutrophils is the release of extracellular vesicles containing proteins and miRNAs, which can target and affect other cells’ status. Finally, neutrophils respond to specific stimuli by forming NETs, which can trigger activation of various immune cells.

The evidence of direct cell-to-cell interaction between neutrophils and other immune cells is scarce with few studies being confirmatory. However, it is established that neutrophils engage interactions with both innate and adaptive immune cells, including DCs, monocytes, macrophages, T cells, B cells, and NK cells (Figure 1), as well as with non-immune cells such as platelets, epithelial and/or endothelial cells [5].

Infection models demonstrate that neutrophils can directly interact with DCs and are capable of capturing and presenting antigens to T cells [38], bridging the innate and the adaptive branches of the immune system [7]. It is well-established that neutrophils and DCs can interact through the binding of Mac-1 (CD11b/CD18) on neutrophils to the C-type lectin DC-SIGN (CD209) on DCs, promoting DCs maturation. Furthermore, the interaction between CEACAM1 on neutrophils and both DC-SIGN and ICAM-1 on DCs plays a critical role in facilitating their communication [16,41,42]. However, direct communication between neutrophils and DCs, has not yet been extensively investigated in T1D. Neutrophils are also thought to directly interact with T cells, promoting their differentiation into Th1 and Th17 subsets [16]. Furthermore, neutrophils can drive the polarization of activated CD4 T cells toward either a Th1 or Th2 response by producing IL-12 or IL-4, respectively [7,43]. Neutrophils are not only pivotal initiators during the early stages of inflammation but also participate in the later stages. They likely play homeostatic roles in both infectious and non-infectious inflammation, interacting with non-immune or somatic cells, such as epithelial cells and platelets [5,7].

### 2.2. Long-Range Signaling Among Neutrophils and Immune Cells via Cytokines, Chemokines, and/or Granule Proteins

As central regulators of innate immunity, neutrophils recruit, activate, and influence other immune cells mainly by secreting a wide array of pro-inflammatory and immunomodulatory cytokines [7,44], or through the release of granule proteins, microvesicles, apoptotic bodies, and/or NETs [5,45]. Importantly, neutrophil granules are generally classified into four groups based on their stages of maturation: primary or azurophil granules (including MPO, proteinase 3 P-R3-, defensins, azurocidin -CAP37-, NE, and cathepsin), secondary or specific granules (comprising lactoferrin, lysozyme, and properdin), tertiary granules (such as those containing arginase and gelatinase), and secretory vesicles, including ectosomes and exosomes [2,46,47]. These molecules (cytokines, granule proteins and microvesicles/apoptotic bodies) in turn, enhance the recruitment and functionality of other immune cells from both the innate and the adaptive systems, such as DCs, B cells, NK cells, CD4 and CD8 T cells, monocytes, and macrophages [2,7,48,49] then triggering a complex pro-inflammatory context. In the pathological context, such mechanism have been clearly demonstrated in multiple autoimmune diseases like RA and SLE [1,29] and could also occur in T1D, potentially in the initial stages of the disease.

Interestingly, neutrophils can be subdivided into multiple distinct subsets based on their transcriptional profiles [50], suggesting that specific neutrophil subpopulations may play critical roles in various pathological contexts, distinct from their traditional role in infection surveillance. For example, a specific subset of neutrophils known as “Low-density granulocytes” (LDGs) has been identified in certain inflammatory contexts and could be the main actor in pathological ones, including T1D. Compared to normal-density neutrophils, these cells exhibit distinct pathogenic phenotypes and functions, producing elevated levels of specific cytokines, including type I interferons, TNF-α, and IFN-γ, contributing to immunostimulatory effects [3,25]. Moreover LDGs exhibit an impaired phagocytic activity, can induce endothelial cytotoxicity by disrupting the differentiation of endothelial progenitor cells into mature endothelial cells, and activate multiple immune cells to produce pro-inflammatory cytokines and chemokines [5,51]. Hence, in T1D, we can hypothesize a multifaceted role for specific neutrophils subsets in initiating pancreatic β-cell destruction and/or dysfunction by producing multiple cytokines, granule proteins or other factors [2,52], all of which could actively be involved in the pro-inflammatory context of the insulitic islet.

#### 2.2.1. Neutrophils-Dendritic Cells Interaction

Neutrophils could be pivotal during T1D initiation and progression in consequence of DCs recruitment through the secretion of CXCL9 and CXCL10 as the latter was observed to be expressed in pancreatic islets in autoimmune diabetes [16,53,54] or other chemokines (CCL2, CCL3, CCL4, CCL5, and CCL20) and pro-inflammatory cytokines (TNF-α and IL-1β) [7,44,46]. Additionally, granule proteins may play a specific role in neutrophils-DCs communication. In fact, neutrophils alarmins, including α-defensins, cathelicidins, and high-mobility group box-1 (HMGB1) granule proteins, act as chemoattractants for DCs [46,55]. Moreover, α-defensins and HNP1 can stimulate pDCs to produce IFN-α [5,56,57]. Importantly, DCs have been shown to contribute to both immunomodulatory effects and triggering of pathogenesis in T1D.

#### 2.2.2. Neutrophils-Monocytes/Macrophages Interaction

It is also well known that neutrophils interact with other innate immune cells, such as monocytes and macrophages [46]. Their interactions are critical in both the initiation and resolution phases of the inflammation and can be instrumental for the initial setting of a pathological context, including T1D. This partnership begins in the bone marrow, where neutrophils interact closely with monocytes and macrophages. Macrophages regulate granulopoiesis by releasing granulocyte colony-stimulating factor (G-CSF) and influence neutrophil preservation through direct cellular contact or CXCL12/CXCR4 signaling [58,59,60]. In the periphery, tissue-resident macrophages play a central role in inflammation by secreting chemoattractants such as CXCL1, CXCL2, and CCL2, as well as pro-inflammatory cytokines like IL-1α, which drive the shift of activated neutrophils to inflamed tissues [46,58,61]. This process is further supported by factors such as G-CSF, GM-CSF, which can prolong neutrophil lifespan [46]. This dynamic interplay has been recognized as a pivotal mechanism for modulating inflammation in a wide range of pathological conditions [62]. Indeed, several studies revealed that pancreatic macrophages and β-cells can produce chemokines, including CXCL1 and CXCL2, recruiting CXCR2-expressing neutrophils from the bloodstream to pancreatic islets [38,63]. Once activated, neutrophils can further release factors that recruit and, in turn, activate other innate immune cells, thereby indirectly promoting the priming of naive antigen-specific T cells [38,64,65]. For example, once in the inflamed tissues, neutrophils can contribute to the recruitment of peripheral blood monocytes by releasing granule proteins such as LL-37 (a cathelicidin), PR3, azurocidin, human neutrophil peptides 1–3 (HNP1–3), and cathepsin G [66,67,68]. Azurocidin and lactoferrin released by neutrophils also drive tissue-resident macrophages toward a pro-inflammatory phenotype [66,69] by enhancing their capacity for phagocytosis and cytokine production (such as TNF-α and IFN-γ) [16,46,67,70].

#### 2.2.3. Neutrophils-B Cells Interaction

An additional noteworthy interaction involves neutrophils and B-cells. In the human spleen’s marginal zone, there is a subpopulation of resident neutrophils, known as B-cell helper neutrophils (NBH cells), which can enhance B-cell proliferation and antibody production by releasing specific cytokines and chemokines [58,71,72]. In this context, neutrophils secrete significant amounts of the cytokines BAFF (B-cell activating factor) and APRIL (a proliferation-inducing ligand), which are essential for B-cell survival, development, proliferation, and plasma cell differentiation [7,16,46,71,73]. Neutrophil-B cell interactions have been observed in various contexts, such as within synovial fluid, where neutrophils produce BAFF, essential for B lymphocyte homeostasis and differentiation [74]. The influence of neutrophils on B cells varies greatly depending on the context, reflecting their versatility and their critical role in preserving immune homeostasis and modulating inflammatory responses [16]. For example, multiple studies revealed that G-CSF and anti-neutrophil cytoplasmic antibodies (ANCA) stimulate neutrophils to release BAFF, highlighting its crucial role in immune regulation [16,73]. B-cell-neutrophil interactions have also been suggested to have a role in the pathogenesis of RA, characterized by the presence of rheumatoid factors and anti-citrullinated protein in patients’ serum. ACPA-positive B cells, when stimulated by citrullinated proteins, secrete CXCL8, a potent neutrophil chemoattractant, which promotes neutrophil migration in vitro [66,75]. Therefore, in light of the increasingly recognized role of B-cells in the pathogenesis of T1D (i.e., in T1D endotypes characterization and their link with the severity of the disease) their intricate interaction with neutrophils may be instrumental to further understand the initiation and progression of T1D.

#### 2.2.4. Neutrophils-T Cells Interaction

Neutrophils are also able to engage with T cells [43]. Activated neutrophils enhance T-cell proliferation and differentiation by releasing cytokines, granules, and NETs [16]. Both in animal models and humans, activated neutrophils have been found to promote T-cell activation, proliferation, and differentiation into effector CD8+ T cells and T helper cell subsets, including Th1 and Th17 cells, thereby supporting adaptive immune responses at sites of inflammation. Activated neutrophils can secrete NE, which modifies CXCL8 into a truncated form, promoting Th17 cell differentiation [16,76], while LL-37 acts as a chemoattractant for CD4+ T cells [5,46,77,78]. Neutrophil-released chemokines such as CXCL1, CXCL7, CCL19, and CCL20 further facilitate T-cell recruitment to these sites [7,44,46]. Finally, neutrophil-derived IL-12 plays a pivotal role in neutrophil-T cell interactions by stimulating T cells to produce IFN-γ. In turn, IFN-γ provides positive feedback to neutrophils, enhancing IL-12 production and amplifying neutrophil activation. This dynamic interplay underscores the versatile role of neutrophils in shaping T-cell responses in both inflammatory and homeostatic conditions [5,79]. However, it remains uncertain whether these immunomodulatory effects arise from pre-existing neutrophils acquiring these properties under specific conditions or from specialized neutrophil subsets that develop in response to distinct triggers [58,60]. Interestingly, neutrophils, often through their differentiation into myeloid-derived suppressor cells (MDSCs), a subset of neutrophils associated with chronic inflammation, can promote the differentiation of CD4+ T cells into Th17 cells, further contributing to the inflammatory milieu [66]. These Th17-mediated responses help to coordinate the activation and regulation of immune functions. Due to their critical roles in autoimmune diseases and resistance to infections, Th17 cells have been extensively studied [34]. These cells are strong neutrophil recruitment inducers, attracting them to inflammation sites via IL-17A and amplifying inflammatory responses [34]. Neutrophils and Th17 cells come together at sites of inflammation through the production of chemokines and cytokines, such as CXCL8, CCL20, CCL22, and IL-17A [34,80]. Notably, Toll-like receptor 8 (TLR8)-activated neutrophils produce IL-23, a key factor driving Th17 differentiation. In vitro studies demonstrated that the supernatant from TLR8-activated neutrophils can promote the differentiation of naive T cells into Th1 cells [34,81]. Specifically, in autoimmune diseases, LDGs promote Th17 cell differentiation and proliferation by responding to IFNs and upregulating costimulatory molecules as well as major histocompatibility complex II (MHC-II) expression [34,82]. For instance, direct interactions between LDGs and T cells have been observed in SLE, where T cell activation by SLE LDGs leads to the release of pro-inflammatory cytokines such as TNF-α and IFN-γ [83,84]. Additionally, IL-17A facilitates neutrophil recruitment to lymph nodes during Th17-mediated immune responses. Recruited neutrophils contribute to these responses by releasing IL-1β, which further supports Th17 cell differentiation [34]. Of note, IL-17A has been demonstrated to play a critical role in the insulitis inflammation thus underscoring the importance of Th17-neutrophils interaction [34,85]. However, neutrophils can also inhibit T-cell activation and differentiation (through DC production of TGF-β, which suppresses T cell proliferation and through inhibiting TLR signaling in DCs) [5,86]. Moreover, activated neutrophils can suppress T-cell responses, through the arginase 1 (ARG1) metabolism. Specifically, neutrophil degranulation promotes the release of ARG1, which reduces local arginine levels. This, in turn, inhibits the actin-binding protein cofilin dephosphorylation in human T cells, thereby disrupting TCR signal transduction and impairing the assembly of CD2 and CD3 during the formation of immunological synapses [16,87]. Neutrophils further inhibit T cells through PD-L1 expression and the promotion of regulatory T cell-like phenotypes via their apoptotic bodies [16,88,89].

The complexity of neutrophil-T cell interactions stems from the activation states of both cell types. For instance, degranulating neutrophils promote T-cell proliferation and differentiation, whereas resting neutrophils suppress T-cell proliferation, activation, and pro-inflammatory cytokine production. Neutrophil-mediated inhibition is mostly effective during the early stages of T-cell activation, while naive and later-stage T cells are less vulnerable to such suppression [16,90].

#### 2.2.5. Neutrophils- NK Cells Interaction

At sites of inflammation, neutrophils interact also with NK cells, through direct cellular contact as well as a complex network of cytokine signaling [16,46,91]. As a matter of fact, it is known that neutrophils play a crucial role in NK cell development and activation, through the release of soluble mediators (such as defensins, NE, and lactoferrin), resulting in an increased NK cells cytokine production and cytotoxicity [16,46,91,92,93]. In contrast, neutrophil-derived granule proteinase G can cleave NKp46, thereby limiting IFN-γ production and degranulation by NK cells [16,94]. This finding suggests a multifaceted role for neutrophils in NK cells activation, depending on the phenotype and subsets of neutrophils involved. Conversely, NK cells can release factors like GM-CSF and IFN-γ which prolong neutrophil survival. This bidirectional interaction highlights the essential role of neutrophils in modulating NK cell responses and fostering immune regulation at sites of inflammation [46]. The precise role of NK cells in the destruction and/or dysfunction of β-cells remains unclear. Several studies have demonstrated that NK cells infiltrate pancreatic tissue in T1D cases, particularly where specific enteroviral infections have been detected [95].

### 2.3. Long-Range Communication Between Neutrophils and Immune Cells via Extracellular Vesicles (EVs), Neutrophils Extracellular Traps (NETs), and microRNAs

#### 2.3.1. The Role of EVs in Neutrophils-Immune Cells Communication

Cells in the body naturally release EVs to facilitate intercellular communication by transferring their contents to other cells [49]. Human neutrophils can release EVs in response to specific activators. These can be classified into ectosomes and exosomes and formation of these vesicles may depend on the assembly of local microdomains in the membranes (endocytic membranes for exosomes and the surface membrane for ectosomes). These microdomains consist of proteins and various types of RNA associated with the cytosolic surface. Exosomes are formed by inward membrane invagination to create precursors (50–150 nm), while ectosomes are formed by outward membrane budding (100–500 nm). Once released into the extracellular fluid, these two types of microvesicles travel to distant tissues to facilitate cell–cell communication. Upon fusion with the plasma membrane of target cells, through endocytosis, these vesicles can influence the physiological processes of the recipient cells, primarily through the delivery of proteins and non-coding RNAs (ncRNAs) [5,16,96,97]. Additionally, ectosomes and exosomes released by neutrophils can play critical roles in pathological conditions, including autoimmune diseases, inflammation, and non-communicable diseases like cancer and Cardiovascular Diseases (CVDs) [5]. Thus, neutrophil-derived EVs can influence disease progression, exhibiting an intrinsic ability to penetrate tissues and interact with target cells [49]. As examples, neutrophil exosomes have been shown to amplify the recruitment of additional neutrophils during inflammation via leukotriene B4 (LTB4) signaling [98]; in autoimmune-associated vasculitis (AAV), neutrophil exosomes, containing autoantigens, can cause endothelial cell damage by increasing apoptosis, reducing proliferation, and impairing wound healing [99]. In RA, neutrophil-derived EVs have been observed to suppress macrophage activation and promote TGF-β release: this, in turn, inhibits macrophages from activating fibroblast-like synoviocytes, which play a critical role in joint damage and disease progression [49]. Finally, it has been recently discovered that human neutrophils can perform trogocytosis, rather than phagocytosis. The effects of trogocytosis between neutrophils and immune-related cells, highlight its role in modulating immune responses [5].

#### 2.3.2. The Role of NETs in Neutrophils-Immune Cells Interactions

Once released by neutrophils, NETs can drive inflammation both directly and indirectly. Increasing evidence suggests that NETs play a critical role in autoimmunity, largely due to their impaired clearance and interactions with other immune cells, including B cells, antigen-presenting cells, and T cells.

In T1D, neutrophils infiltrate the pancreas and release NETs, even in presymptomatic individuals and elevated levels of NE and PR3 are strongly associated with increased NET formation [25]. Furthermore, circulating levels of NE and PR3 are positively correlated with the presence of β-cell specific autoantibodies [16,33]. The nucleic acids present in NETs can stimulate pattern recognition receptors, driving cytokine production from various cell types. Moreover NETs carry immunostimulatory proteins activating other immune and stromal cells, and thus stimulating the type I interferon pathway and the NLRP3 inflammasome [3,100]. The process of NETosis promotes also the recruitment of more neutrophils, which intensifies autoimmune responses [5]. NETs can also activate other immune cells, such as monocytes and macrophages, to secrete IL-1β through the NLRP3 inflammasome, thereby sustaining a pro-inflammatory environment [3,100]. The interaction between the inflammasome and NETs has been extensively documented in a variety of diseases. The inflammasome is a multi-protein complex activated in response to inflammation and immune-related challenges. It is primarily found in macrophages, neutrophils, DCs, and other immune and inflammatory cells, playing a key role in inducing inflammatory and immune responses by recognizing pathogen-associated molecular patterns (PAMPs) and danger-associated molecular patterns (DAMPs) [101,102,103]. The inflammasome has been shown to play a significant role in the pathogenesis and progression of various diseases, including CVDs, metabolic diseases, cancer and autoimmune disorders [101,104]. Recent studies have highlighted the central role of NETs in activating the NLRP3 inflammasome in macrophages [100,105]. The NLRP3 inflammasome is an intracellular macromolecular complex that recognizes danger signals and triggers an inflammatory response, primarily through the release of IL-1β and IL-18. IL-18 can trigger NET release and enhance caspase-1 activation in macrophages through a feed-forward loop. As a result, NETs induce the synthesis of IL-1β and IL-18 in macrophages, which in turn promotes NET formation in neutrophils [100,106]. Enhanced NETosis and the release of granule proteins further promote pathogen phagocytosis by macrophages [58,107,108]. In the context of diabetes, limited data exist on the relationship between NETs and the inflammasome [100]. In the marginal zones of the spleen neutrophils, can also release NET-like structures along with cytokines, furthering antibody production in activated B cells, in response to signals from sinusoidal endothelial cells [7,72]. An indirect interaction between NETs and T cells was observed in the joints of a RA patient [109] as fibroblasts presented citrullinated peptides associated with the NETs. However, NETs seem to directly prime CD4+ T cells, interacting with TCR, improving the T cell response to specific antigens and suboptimal inducements [83,110], as well as enhancing the release of Th17-promoting cytokines, and consequently the differentiation of Th17 cells; this process would seem to be further exemplified by cathelicidin, a peptide secreted by neutrophils during NET formation [34,78,111]. As a matter of fact, this peptide drives CD4+ T cells to Th17 differentiation rather than a Th1 profile, and enhances Th17 cell survival by inhibiting apoptosis [16]. Additionally, NETs can stimulate IFN-α production by DCs, and thereby the generation of CD4+ and CD8+ T cells, which through the releasing of IFNγ, may act as a key link between innate and adaptive immune responses in the pathogenesis of T1D [16,35]. A study conducted on early postnatal NOD mice suggests that NET-induced activation of DCs leads to the polarization of T cells towards a Th1 response, although the exact underlying mechanism remains to be fully elucidated [35,38].

#### 2.3.3. The Role of Secreted miRNAs in Neutrophils-Immune Cells Interactions

MicroRNAs (miRNAs) are been suggested to be key modulators of the islet inflammation in T1D [112] and serve as a communication module between immune system and pancreatic endocrine cells [113], as well as between NETs and cells. During and after NETosis, neutrophils express several miRNAs targeting other cells known as NET-miRNAs [114]. Notable NET-miRNAs include miRNA-142-3p, which enhances TNF-α production in macrophages [7,115], and the miR-17/92 cluster, which targets monocytes [114]. In addition to miRNAs synthesized by neutrophils to regulate NETosis, miRNAs can also be expressed by other cells in response to NETosis, as the development of NETs may influence changes in the expression levels of various miRNAs in neighboring cells [114]. There are miRNAs produced by macrophages, endothelial cells, and platelets, that are transported through exosomes in the bloodstream to interact with neutrophils and target genes involved in the regulation of NETosis. Exosomal miRNAs have been identified in four studies, which revealed that five different miRNAs are secreted by various cell types to regulate NETosis. These miRNAs include miR-142a-3p, miR-15b-5p, miR-378a-3p, miR-505, miR-4512, and miR-125a-3p [114]. Furthermore, a recent study showed that high levels of miR-146a were contained in exosomes derived from macrophages activated with oxidized low-density lipoproteins (ox-LDLs); the authors demonstrated that co-culturing these exosomes with neutrophils promoted NET formation [116,117].

## 3. HYPOTHESIS-2: Neutrophils Can Act as Antigen Presenting Cells Triggering Islet Autoimmunity

**Figure 2:**
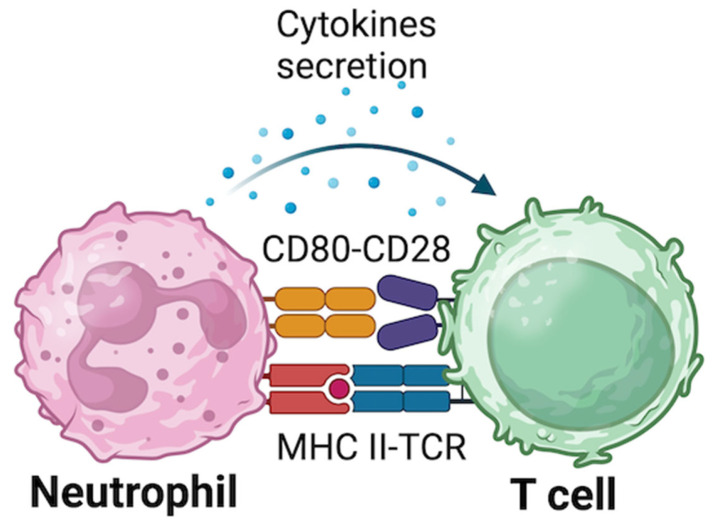
Hypothesis-2: neutrophils act as antigen presenting cells using a three-signals model that is able to prime and expand antigen specific T cells triggering or exhacerbating autoimmunity. Signal 1 consists of the interaction between MHC-peptide complex and TCR; this signal alone cannot activate APCs. Signal 2 is based on the interaction of costimulatory molecules, expressed on APCs, such as CD80 or CD86, with other molecules expressed on T cells, such as CD28. Signal 3 consists of the cytokines secretion by activated APCs inducing the differentiation of CD4+ T cells.

Antigen presenting cells (APCs) are a class of cells able to internalize and/or process extracellular or intracellular antigens; products of this process are then presented by surface proteins called mayor histocompatibility complex (MHC) to be recognized by CD4+ T cells [118]. While all nucleated cells are APC, only some of them, called professional APCs, can respond to the three-signals model that is able to prime and expand antigen specific T cells [119]. This model is based on 3 different signals that are crucial for their activity. Signal 1 consists of the interaction between MHC-peptide complex and T cell receptor (TCR); this signal alone cannot activate APCs. Signal 2 is based on the interaction of costimulatory molecules, expressed on APCs, such as CD80 or CD86, with other molecules expressed on T cells, such as CD28. Signal 3 consists of the cytokines secretion by activated APCs inducing the differentiation of CD4+ T cells (Figure 2) [120]. Well known professional APCs are DCs, macrophages and B cells; DCs and macrophages internalize pathogens and cellular debris by phagocytosis [121,122,123], while B cells use the B cell receptor to internalize antigens [124]. After the uptake, antigens are processed into the cells and peptides are presented to T cells that can be committed to become helper or cytotoxic cells and produce memory cells.

Several evidence have highlighted that neutrophils can contribute to adaptive immunity by transporting and presenting antigens to T cells [8,125,126]. Resting neutrophils do not express MHC class II or costimulatory molecules and are unable to stimulate naïve CD4+ T cells [8,126], this means that resting neutrophils are not professional APCs. However, during pro-inflammatory stress neutrophils can express MHC class II or costimulatory molecules, such as CD80 or CD86 [127]. Different studies have been demonstrated CD80 and CD86 are stored in cytosol of resting neutrophils and of interest also the mRNAs for the synthesis of these proteins have been detected in the cytoplasm of resting neutrophils, excluding the possibility to have been detected the protein due to the ability of the cell to uptake proteins from the environment [128,129]. mRNA for MHC class II was also detected in these neutrophils suggesting that its expression is regulated by post-transcriptional modification [130,131]. Interestingly, when neutrophils were activated in vitro, mRNAs levels increase and cytoplasmatic CD proteins decreased due to their rapid translocation onto the cell surface [132,133]. Sandilands et al. detected MHC class II molecules in 10% of resting neutrophils [132]. However, for the activation of T cells, a specific number of MHC class II complexes must be present; in particular, 200–300 MHC class II complexes capable of binding to T cells are required to initiate their activation [134].

Neutrophils can be stimulated to express stimulatory and co-stimulatory molecules giving them the ability to be APCs. The treatment of neutrophils with IFN-γ, IL-3 or GM-CSF is sufficient to induce MHC class II expression. Notably, it has been demonstrated that neutrophils isolated from peripheral blood and cultured with antigens and IFN-γ, in combination, become able to express MHC class II presenting those antigens [135]. While some studies suggested that neutrophils expression of MHC class II is a consequence of monocytes contamination during isolation procedure from peripheral blood, additional research showed that highly purified neutrophils maintain this ability [136,137]. However, in vitro cytokine stimulation may not accurately reflect the complex in vivo microenvironment, potentially leading to an overestimation of neutrophil APC capabilities [2].

Interestingly, neutrophils treated with a specific set of cytokines acquire a hybrid cellular phenotype known as neutrophil-DC cell. This specific phenotype is characterized by the expression of some neutrophils markers, such as CXCR2 and CD16, and some DCs markers, such as MHC class II, CD80 and CD86, thus being able to present antigens to the adaptive immune system [138,139].

Neutrophils can acquire antigen-presenting ability in the presence of antigen-specific memory T cells and antigens, even without stimulation by exogenous cytokines. This process occurs during adjacent APCs antigen presentation, leading to the activation of antigen-specific T cells. In turn, these T cells produce cytokines, primarily IFN-γ, which subsequently drive the differentiation of neutrophils. This phenomenon is observed in the presence of antigen-specific memory T cells but not naïve T cells; indeed, memory T cells exhibit higher IFN-γ production and require lower levels of costimulatory molecules for activation, and display enhanced production of IFN-γ. Furthermore, evidence suggests that memory T cells constitutively express intracellular mRNA for IFN-γ, indicating a “pre-activated state”. These studies suggest the crucial role played by antigen-specific T-cell in combination with exposure of neutrophils to specific antigens and cytokines. Furthermore, as just observed in neutrophils treated with cytokines, co-cultured neutrophils with antigens only can’t induce the expression of MHC class II [135].

Overall, considering the role of neutrophils as antigen-presenting cells, it is plausible to hypothesize a specific involvement in antigen presentation during multiple stages of T1D, both at the onset of the disease and throughout its progression. Several studies support the hypothesis that neutrophils act as APCs in the initiation and modulation of immune responses. For example, in mice vaccinated with BCG, neutrophils have been observed to internalize antigens at the injection site and subsequently present them to T cells within the T cell zone of draining lymph nodes [140]. This neutrophil-mediated antigen presentation has also been demonstrated in vaccinated primates where neutrophils constitute the main cell population responsible for antigen internalization [141]. In a mouse model of inflammatory bowel disease, isolated neutrophils from inflamed colon lesion expressed high levels of MHC II and CD86, and were able to present ovalbumin antigen to naïve CD4+ T cells in an MHC II-dependent process [142]. Driven by inflammatory chemokines, these cells may infiltrate inflamed draining lymph nodes, where could bolster protective immunity by initiating antigen-specific CD4+ and CD8+ T cell responses, thereby providing a preemptive defense against secondary infections. Conversely, the premature activation of cytotoxic CD8+ T cells might exacerbate systemic inflammation, potentially culminating in tissue injury and organ dysfunction. Although APC-like neutrophil differentiation primarily occurs within infected tissues, severe inflammatory states, such as sepsis, may result in their systemic dissemination, making them detectable in peripheral blood [143]. Furthermore, the presence of neutrophils exhibiting elevated levels of MHC class II and co-stimulatory molecules has been identified in individuals with Wegener’s granulomatosis and rheumatoid arthritis [144,145], suggesting a critical role for activated neutrophils in the pathogenesis of autoimmune diseases, including T1D. The clinical significance of neutrophil APC function in T1D pathogenesis remains to be fully elucidated, and more comprehensive studies are needed to validate these findings.

## 4. HYPOTHESIS-3: Neutrophils Can Release Factors That Are Involved in the Direct Damage of the β-Cells

Upon inflammatory insults, neutrophils are able to release various factors, including ROS, proteolytic enzymes and/or NETs [146], which can directly damage surrounding cells, including β-cells. These molecules, especially ROS, have been shown to contribute to oxidative stress, inflammation, and structural disruption within the islets [147], thereby potentially exacerbating β-cell destruction and impairing their functionality in the context of T1D [25,148].

In the pancreatic tissue of islet AAb+ and T1D donors, neutrophils are increased and predominantly located in the exocrine portion of the pancreas, likely associated with the microvasculature or interspersed within the acinar tissue [25]. This increase is particularly evident in donors with new-onset T1D compared to those with long-standing disease, suggesting that an active inflammatory process is required to chemoattract neutrophils to the pancreas. Notably, an increased number of neutrophils has also been observed in AAb+ donors, although this increase is far less pronounced than in new-onset donors. Interestingly, in new-onset T1D donors (<9 weeks from diagnosis—DiViD cohort), some neutrophils have been observed in close proximity to pancreatic islets, although the evidence remains sparse, in part due to the limited availability of studies and number of new-onset donors analysed. Nevertheless, this observation suggests that the increase in neutrophils in pancreatic tissue and their localization near pancreatic islets could be a phenomenon strictly confined to the initial phase of stage 3 T1D or to the stages immediately preceding disease onset—both periods that are difficult to capture immunohistologically. Importantly, in pancreas of NOD mice, infiltrating neutrophils peaked at 3–4 weeks of age (long before the onset of the disease in this animal model) while decreasing thereafter, demonstrating that the major increase of these cells is moderately transient during the progression of the disease; moreover, these are tightly associated with β-cells and mediated the activation of plasmacytoid dendritic cells [22,149]. These data suggest that neutrophils, both in human and in animal models, are present in the vicinity of pancreatic islets and also associated with β-cells, thus providing the inflammatory cues needed for their subsequent damage and death.

Exposure of neutrophils to a pro-inflammatory environment, such as that present in the pancreas during an early insulitic process, may stimulate neutrophils to release ROS. These ROS, including superoxide and/or hydrogen peroxide can also target highly susceptible β-cells either through direct exposure or via release into the microvasculature near the pancreatic islets, thereby contributing to β-cell damage and dysfunction [150,151]. Notably, neutrophils-induced oxidative stress has been previously shown to cause tissue damage in various organs and under diverse pathological conditions [152], thus potentially including also pancreatic islets during T1D progression. It is also important to note that β-cells are known to be more susceptible to ROS and oxidative stress compared to alpha cells, primarily due to their lower intrinsic antioxidant capacity. This heightened vulnerability is associated with reduced levels of antioxidant enzymes, limiting their ability to efficiently counteract ROS-induced damage [153,154,155]. Such differential susceptibility may explain, at least in part, the preferential β-cell dysfunction and loss observed in the early stages of T1D pathogenesis [156].

Granule-associated enzymes have also cytotoxic potential. As a matter of fact, the release of neutrophils’ granules has been associated to the infiltrated tissue damage in multiple contexts [157,158,159]. Degranulation has also been reported to be associated with hypoxia [160], a condition present also at the level of pancreatic islets in diabetes [161,162], or involving pancreatic microvasculature, thus exacerbating the tissue damage. Of note, such damage is particularly associated with the activity of specific enzymes which may function as tissue-digesting enzymes thus damaging the surrounding tissue causing direct cell death and/or exacerbating the inflammation [26].

Additionally, NETs can further exacerbate local tissue damage. NETosis, is often triggered by inflammatory stimuli and can directly contribute to β-cell by physical interaction with the islet cells. As a matter of fact, it has been demonstrated that extracellular histones (one of the main component of NETS) can damage human islets in-vitro [163]. Moreover, proteolytic enzymes such as elastase and MPO, released during NETosis, have also been reported to degrade extracellular matrix components and cellular structures [164], potentially compromising β-cell survival.

**Figure 3:**
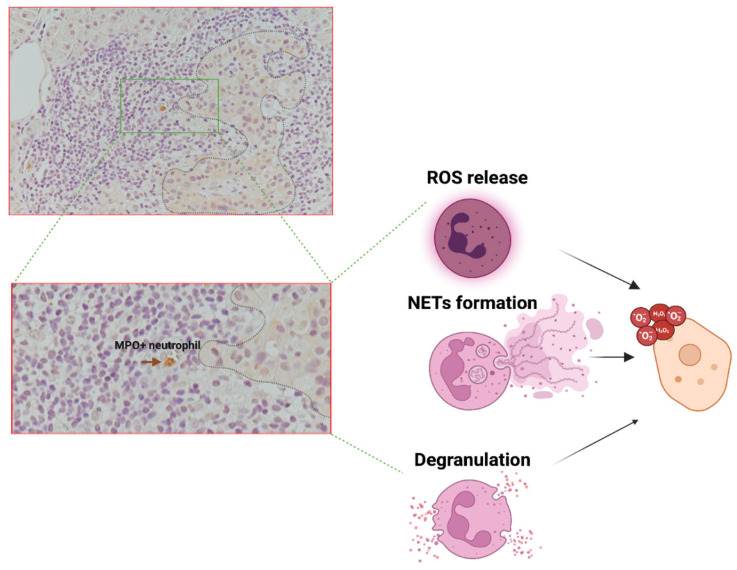
Hypothesis-3: neutrophils are found in pancreatic islets where can damage β-cell through ROS release, degranulation and NETs formation. Pro-inflammatory environment may stimulate neutrophils to release ROS, such as superoxide and hydrogen peroxide, that directly target β-cells. Granules released by degranulation have a cytotoxic activity, causing a direct cell death and/or exacerbating the inflammation. NETosis, frequently induced by inflammatory stimuli, can directly impact β-cells through physical interaction with islet cells; proteolytic enzymes such as elastase and MPO, released during NETosis, have been observed to degrade extracellular matrix components and cellular structures, potentially jeopardizing β-cell survival. Immunohistochemistry image of DIVID #3 donor (34 y.o, female) adapted from [25].

Overall, neutrophils infiltrating the pancreas in early stages or in the first period of Stage 3 T1D, may directly contribute to pancreatic islets and β-cell damage in multiple ways (Figure 3) thus representing one of the putative mechanisms involving neutrophils in T1D pathogenesis. However, we emphasize that this hypothesis remains highly debated, primarily due to the scarce evidence of neutrophil infiltration closely associated with pancreatic islets in donors with T1D. We propose that an extensive and in-depth analysis of infiltrating neutrophils in the pancreas of donors with stage 2 T1D should be conducted, along with the identification of additional specific circulating biomarkers of neutrophil activation and infiltration (i.e., circulating small RNAs), in order to define a precise role and timing for neutrophils in the early pathogenesis of T1D.

Finally, we cannot exclude that intrinsic, potentially genetically driven early β-cell stress and dysfunction [156,165] may initially attract and activate neutrophils, which could subsequently cause excessive damage and inflammation, thereby triggering the autoimmune response in susceptible individuals.

## 5. Conclusions and Future Perspectives

Neutrophils, as key effectors of the innate immune system, engage in complex bidirectional interactions with both innate and adaptive immune cells, shaping immune responses in both physiological and pathological contexts. These interactions are particularly relevant in autoimmune diseases, where neutrophils contribute to both the initiation and regulation of inflammation. Of interest, neutrophils can perform highly diverse functions, and understanding the precise role these cells play in either physiological or pathological conditions is a challenge. Indeed, neutrophils represent a highly heterogeneous population, both in terms of their composition and their functionality and several evidence indicates that different neutrophil subtypes can have crucial roles in the development, progression or resolution of various diseases, including sepsis, SLE, rheumatoid arthritis, multiple sclerosis, type 1 diabetes and cancer [166,167,168,169].

In the context of T1D, accumulating evidence from both animal models and human studies supports the notion that neutrophils play a pivotal role, particularly during the early phases of disease development.

In this review, we have delineated how neutrophils communicate with immune cells such as dendritic cells, macrophages, B cells, T cells, and NK cells through a “remote and wireless” network of signaling pathways involving cytokines, chemokines, granule proteins, EVs, NETs, and microRNAs. Additionally, neutrophils may function as antigen-presenting cells and contribute to β-cell destruction through the release of ROS and proteolytic enzymes, further fueling the pro-inflammatory milieu of the pancreatic islets. These mechanisms, which are not mutually exclusive, collectively promote oxidative stress, immune activation, and β-cell dysfunction, ultimately driving the autoimmune process in T1D. Furthermore, the contribution of neutrophils to organ alterations beyond the pancreas in T1D pathogenesis remains unexplored.

It would be also interesting to investigate in detail the potential role of insulin in influencing neutrophil functions in T1D etiopathogenesis. Specifically, exploring its role in regulating protein production by neutrophils is particularly relevant, as an immune response relies also on the production and secretion of a wide range of proteins, including cytokines.

Despite significant advancements in understanding the multifaceted role of neutrophils in T1D, many questions remain unanswered. The emerging view of neutrophils as highly interactive “social network” cells underscores the need for further research to dissect their functional heterogeneity, their crosstalk with other immune populations, and their full repertoire of effector molecules. Unraveling these complexities could provide novel insights into the immunopathogenesis of T1D and open new avenues for therapeutic interventions aimed at modulating neutrophil activity in autoimmune diabetes.
